# Rediscovery of viomellein as an antibacterial compound and identification of its biosynthetic gene cluster in dermatophytes

**DOI:** 10.1128/aem.02431-24

**Published:** 2025-04-08

**Authors:** Akihiro Ninomiya, Keisuke Masuda, Tsuyoshi Yamada, Misa Kuroki, Sayaka Ban, Takashi Yaguchi, Syun-ichi Urayama, Daisuke Hagiwara

**Affiliations:** 1 Faculty of Life and Environmental Sciences, University of Tsukuba 623469, Tsukuba, Ibaraki, Japan; 2 Graduate School of Agricultural and Life Sciences, The University of Tokyo 13143, Bunkyo, Tokyo, Japan; 3 Graduate School of Life and Environmental Sciences, University of Tsukuba 98393 , Tsukuba, Ibaraki, Japan; 4 Teikyo University Institute of Medical Mycology, Hachioji, Tokyo, Japan; 5 Asia International Institute of Infectious Disease Control, Teikyo University 13094, Itabashi, Tokyo, Japan; 6 Medical Mycology Research Center, Chiba University 118076 , Chiba, Chiba Prefecture, Japan; 7 Microbiology Research Center for Sustainability, University of Tsukuba 623473, Tsukuba, Ibaraki, Japan; Royal Botanic Gardens, Surrey, United Kingdom

**Keywords:** *Trichophyton rubrum*, secondary metabolite, antibiotic, biosynthetic gene, naphthopyranone, heterologous expression

## Abstract

Fungi produce a wide variety of compounds, especially those that exhibit biological activity. Such compounds may aid the survival of fungi in the environment or may contribute to host infection for pathogenic species. Regarding dermatophytes, which affect a large number of patients worldwide, studies on metabolites that exhibit biological activity are scarce. In this study, to gain insight into the interaction with skin microbiota at the site of infection, we searched for compounds that exhibit antibacterial activity among the metabolites of *Trichophyton rubrum*. We rediscovered viomellein, a red pigment, as a potent antibacterial compound and identified its biosynthetic gene (*vio*) cluster by RNA-sequencing and gene deletion analyses. Sequential reconstruction of the *vio* cluster genes in *Aspergillus oryzae* revealed the biosynthetic pathway for viomellein via nor-toralactone, semivioxanthin, and vioxanthin production. The *vio* gene cluster is widely conserved among dermatophytes and is also present in some *Aspergillus* and *Penicillium* species. Consistent with the results, viomellein and its structural analogs, xanthomegnin, and vioxanthin, were shown to be produced by most dermatophyte species. These results suggest that dermatophytes can produce diverse naphthopyranone compounds, some of which have strong growth inhibitory effects against bacteria. This study provides a previously unknown molecular entity for antibiotic production by dermatophytes and provides insight into the interaction between commensal bacteria and dermatophytes.

**IMPORTANCE:**

Dermatophytes are widespread human pathogens in the world, but the mechanisms of infection have been little studied. Although bacterial density at the site of infection is abundant, interaction between dermatophytes and the bacterial community has not yet been studied. Here, to understand the infection ecology of dermatophytes, we searched for antimicrobial substances that would be effective against the dermal bacterial community. We discovered viomellein, which exhibits strong antibacterial activity against gram-positive bacteria such as *Staphylococcus aureus*, and its biosynthetic genes are shared not only by dermatophytes but also by other fungi. Since many dermatophytes showed the ability to produce viomellein, it is likely that this is the initial infection strategy of dermatophytes, which has been a mystery for long.

## INTRODUCTION

Dermatophytes are the main cause of infection on the skin and nails, affecting more than one billion people worldwide (1). These fungi are characterized by their special ability to degrade the keratin layer and thus invade keratinized skin tissues in humans and animals. The major pathogenic dermatophytes that infect human skin are *Trichophyton rubrum* and *Trichophyton tonsurans*, while *Trichophyton benhamiae* and *Microsporum canis* are zoophilic and can infect guinea pigs, cats, and dogs. During infection, inflammatory activity against the invaders in the host is relatively low (2), and pigment produced by the pathogens is sometimes visible around the infection site. Although the clinical manifestations and macroscopic features of dermatophyte infection have been defined, the physiology and biology of dermatophytes have been poorly studied at the molecular level.

Recent studies have revealed that the human skin microbiota is complex and is shaped by a variety of microorganisms, including bacteria, fungi, and viruses (3). The composition of the skin microbiota is variable among individuals and diverse between different anatomical sites (4, 5). Regarding sites on the foot, 1.04 × 10^7^ colony-forming units (cfu)/cm^2^ skin have been counted in the toe cleft and 4.08 × 10^5^ cfu/cm^2^ skin in the sole (6). Bacteria of the family *Staphylococcaceae* are ubiquitous on healthy foot skin. The skin microbiota can be affected by environmental factors, such as moisture, sweat (i.e., chemical composition), and skin condition (7). However, little is known regarding the ecological role of commensal bacteria in the dermatophyte infection process. In particular, the chemical and physical interactions between commensal bacteria and dermatophytes during infection remain poorly understood.

An early report demonstrated that *Trichophyton mentagrophytes* produced penicillin-like antibiotics *in vitro* (8). The production of penicillin-like substances has since been reported in dermatophytes by several studies (9–11). *Microsporum cookei* was also shown to inhibit the growth of *Staphylococcus aureus* strains that were resistant or susceptible to benzylpenicillin, suggesting the production of non-penicillin antibiotics (12). Streptomycin-like activity was also demonstrated by Youssef et al*.* 10, in which 32 clinical isolates of several dermatophytes were investigated, and benzylpenicillin and streptomycin activities were detected in the liquid culture filtrates of 24 isolates. From these studies, it was proposed that dermatophytes are able to produce antibiotics, including β-lactam and other types of compounds. However, the chemical basis for the generation of antibiotic compounds has not been reported to date. Recently, the above observations have been supported by genomic evidence that the penicillin biosynthesis gene cluster is widely conserved among dermatophyte species (13, 14). Direct genetic evidence for antibiotic production is yet to be reported but would provide insight into microbial interactions at the infection site.

Besides the above-mentioned antibiotic compounds, little is known about secondary metabolites produced by dermatophytes, with the only examples being neosartoricin B, xanthomegnin, and related compounds. Neosartoricin B was discovered by heterologous expression of the biosynthetic gene cluster of *T. tonsurans* in the host *A. nidulans* (15). Although the genetic basis for the compound was reported, the presence of the compound in the culture filtrate of this fungus was not confirmed. The production of xanthomegnin and related compounds was originally identified in the 1960s in *Trichophyton megnini*, *T. rubrum*, and *Trichophyton violaceum* (16–18). Xanthomegnin was regarded as a mycotoxin since it showed genotoxicity (19) and a deconjugating effect on rat liver mitochondria (20). Xanthomegnin also has an inhibitory effect on inducible nitric oxide synthase activity, suggesting that secreted xanthomegnin can influence the immune response of host cells (21). Since xanthomegnin was frequently detected in clinical skin and nail samples infected by *T. rubrum* (22), this compound might play a key role in the establishment of dermatophyte infection. However, genetic characterization of xanthomegnin production is lacking and has prevented its verification.

In the present study, we revisited antibiotic compounds produced by dermatophytes, which are essential for understanding the ecological resilience of the dermal microbiota during infection. Viomellein was rediscovered as the main antibacterial pigment compound, and the biosynthetic gene cluster was characterized. These findings provide a previously unknown molecular entity for antibiotic production by dermatophytes and offer insight into the interaction between commensal bacteria and dermatophytes.

## RESULTS

### Non-penicillin antibiotic activity exhibited by dermatophytes

**Fig 1:**
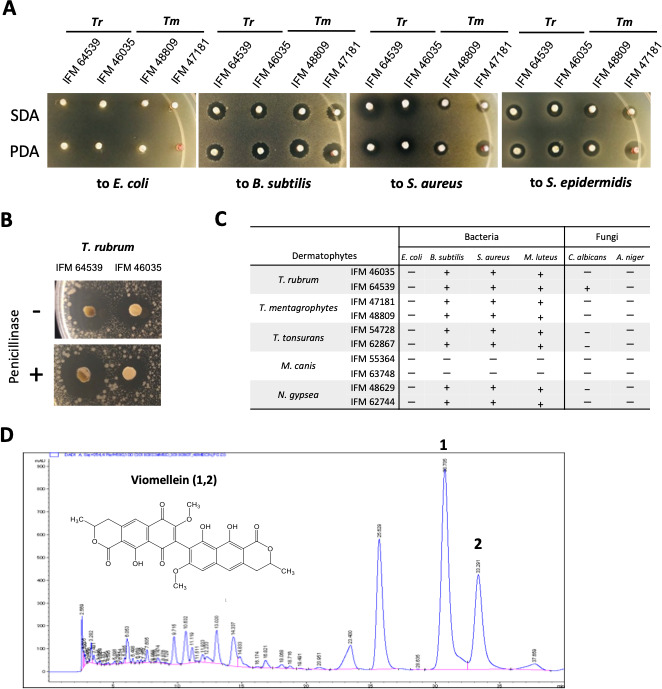
Antimicrobial activity test of dermatophytes. (**A**) Diffusion assay using an agar plug against bacteria. *T. rubrum* (Tr) and *T. mentagrophytes* (Tm) strains were grown on SDA and PDA at 30°C for 7 days, and the agar plugs (diameter: 5 mm) were placed on LB agar containing precultured bacteria. The test plates were incubated at 30°C for 1 day. (**B**) Penicillinase assay. The agar plugs of *T. rubrum* on PDA were placed on LB agar containing *B. subtilis* with and without penicillinase. The test plates were incubated at 30°C for 1 day. (**C**) Paper disc assay against bacteria and fungi. *T. rubrum*, *T. mentagrophytes*, *T. tonsurans*, *M. canis*, and *N. gypsea* strains were grown on SDA at 30°C for 3 weeks. The methanol extracts of each lyophilized strain were examined for growth inhibitory effects against *E. coli*, *B. subtilis*, *S. aureus*, *M. luteus*, *C. albicans*, and *A. niger*. The plates were incubated for 1 or 2 days. “+” indicates halo formation, whereas “−” indicates no halo formation around the paper disc. (**D**) HPLC analysis of the culture extract of *T. rubrum. T. rubrum* was cultured in SDA for 3 weeks at 30°C. The culture plates were lyophilized and extracted with methanol and then ethyl acetate. The extracts were combined, evaporated, and resuspended with ethyl acetate and water. After separation, the ethyl acetate phase was collected, concentrated *in vacuo*, and analyzed by HPLC. The chemical structures of compounds **1** and **2** are shown.

The strains of *T. rubrum* and *T. mentagrophytes* showed orange to red pigmentation on sabouraud dextrose agar (SDA) and potato dextrose agar (PDA) (Fig. S1). In a diffusion assay using agar plugs, these strains showed an inhibitory effect against *Bacillus subtilis*, *S. aureus*, and *Staphylococcus epidermidis*, but not against *Escherichia coli*, suggesting the production of antibiotic compounds that target gram-positive bacteria (Fig. 1A). The antibiotic activity was retained even in the presence of penicillinase, which indicated that the activity was not attributed to compounds with beta-lactam rings, such as penicillin (Fig. 1B). Then, we examined cell extracts of *T. rubrum*, *T. mentagrophytes*, *T. tonsurans*, *Nannizzia gypsea*, and *M. canis* by a paper disk assay. All of the microbial cell extracts tested, with the exception of *M. canis*, showed an inhibitory effect against gram-positive bacteria, but most showed no antifungal activity (Fig. 1C).

### Identification of viomellein and related compounds

We set out to identify the compounds showing antibiotic activity. *T. rubrum* IFM 64539 was cultured on SDA, and the whole culture was extracted by organic solvents. Based on the antibacterial activity against *B. subtilis*, the extract was fractionated (Fig. S2), and two antibacterial red pigments (**1**, **2**) were obtained (Fig. 1D). By high-resolution electrospray ionization mass spectrometry (HRESIMS), the molecular formulae of compounds **1** and **2** were determined as C_30_H_24_O_11_, which is consistent with a fungal pigment viomellein (23). Then, we compared the retention times of compounds **1** and **2**, and commercially obtained viomellein standard in LCMS analysis (Fig. S3). As a result, the standard yielded two peaks, and the retention times of the former and the later peak matched with those of compounds **1** and **2**, respectively. Therefore, compounds **1** and **2** were identified as viomellein. In the viomellein molecule, two naphthopyranone moieties are linked via a single C-C bond. Its structure is suggestive of atropic isomerism, which may explain the two distinct peaks in the LCMS analysis.

### Identification of the biosynthetic gene for viomellein

To identify the biosynthetic genes for viomellein, we first collated all of the secondary metabolism core genes (PKS, NRPS, and PKS-NRPS hybrid) from the genomes of *T. rubrum*, *T. mentagrophytes*, *T. tonsurans*, *M. canis*, and *N. gypsea*. In total, 28–44 genes were detected in each species, among which 16 genes (6 for PKS; 7 for NRPS; and 3 for the PKS-NRPS hybrid) were shared among all species (Table S1). Interestingly, the secondary metabolism genes of *T. rubrum* and *T. mentagrophytes* were highly conserved, with all *T. rubrum* gene sequences showing high homology with those of *T. mentagrophytes*.

**Fig 2:**
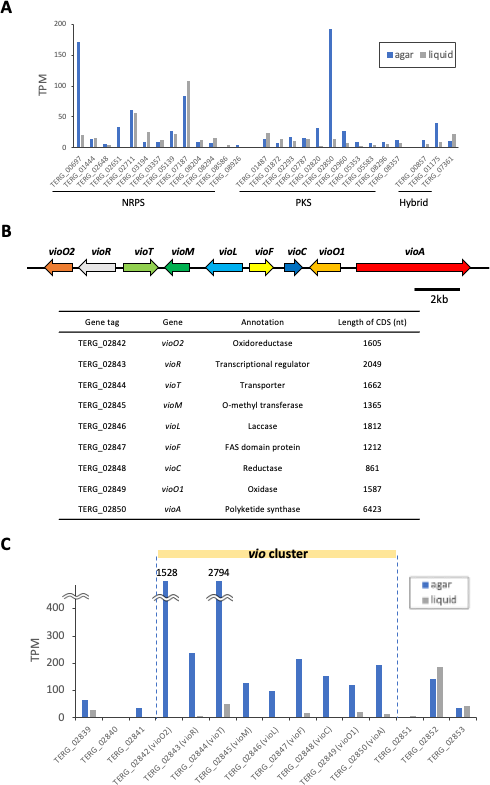
Expression level of the *T. rubrum* secondary metabolism core genes and *vio* cluster genes. (**A**) *T. rubrum* was cultured on SDA for 9 days and in SDB for 12 days. The RNA was purified and transcriptome analysis was performed. The transcripts per million (TPM) were calculated, and the TPM for the secondary metabolism core genes, which encode NRPS, PKS, and the hybrid enzyme, are shown. (**B**) Biosynthetic gene cluster for viomellein (*vio*). (**C**) The TPM for the *vio* genes are shown.

To identify the core gene responsible for viomellein production, we conducted a comparative transcriptome analysis in *T. rubrum*. The expression level of each core gene was compared between agar plate and liquid cultures, where the fungus was able and unable to produce viomellein, respectively. Under conductive conditions (growth on SDA), four genes, TERG_00697, TERG_02711, TERG_07187, and TERG_02850, showed a relatively higher (>50) transcripts per million (TPM) (Fig. 2A). The expression level of TERG_02711 and TERG_07187 was also high in liquid culture, thus we omitted them from the candidates. From a structural perspective, viomellein is predicted to contain a polyketide backbone, and we thus considered TERG_02850 as the most likely potential candidate gene for the biosynthesis of the viomellein backbone. AntiSMASH predicted that the biosynthetic gene cluster within TERG_02850 consists of nine genes including oxidoreductase, transcription factor, transporter, O-methyltransferase, laccase, and unknown function genes (Fig. 2B). The expression levels of these genes were coordinately induced in agar culture, compared to liquid culture (Fig. 2C), which suggested that the cluster is involved in viomellein biosynthesis. We named these genes: *vioO2*, *vioR*, *vioT*, *vioM*, *vioL*, *vioF*, *vioC*, *vioO1*, and *vioA* (TERG_02850), following the previous study (24).

### Verification of the viomellein biosynthetic gene by deletion mutation

**Fig 3:**
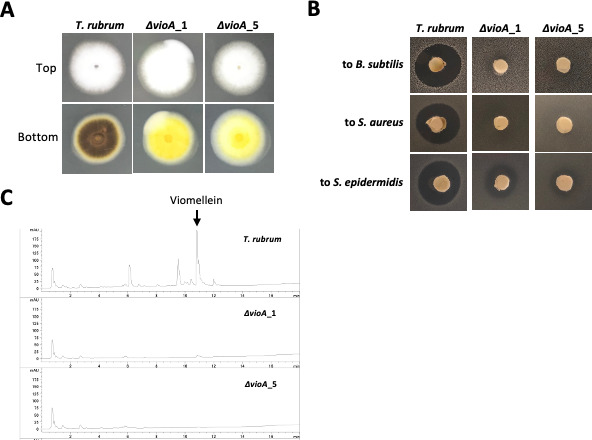
Colony appearance and metabolite analysis of the *vioA* mutants. (**A**) The parental strain and the *vioA* deletion mutants were grown on SDA at 30°C for 9 days. (**B**) Diffusion assay using an agar plug. *T. rubrum* (parental strain) and the mutant strains were grown on SDA at 30°C for 7 days, and the agar plugs (diameter: 5 mm) were placed on the LB agar containing precultured bacteria. The test plates were incubated at 30°C for 1 day. (**C**) HPLC chart of the extracts from the parental and mutant strains.

To determine if the *vio* cluster is responsible for viomellein production, we constructed a deletion mutant of the *vioA* gene in the *T. rubrum* IFM 46035 strain using the CRISPR/Cas9 system. We successfully obtained two independent mutants, which were verified by genomic PCR and Southern hybridization analyses (Fig. S4). Colonies of the mutant strain defective in *vioA* showed no dark brown pigmentation on PDA, but the colony growth rate was comparable to that of the parental strain (Fig. 3A). An agar-plug assay revealed that the *vioA* mutant had no activity or weakened activity against *B. subtilis*, *S. aureus*, and *S. epidermidis* (Fig. 3B). The metabolite profile of the mutant strains was investigated and confirmed the lack of viomellein production (Fig. 3C). These results suggested that *vioA* is essential for viomellein biosynthesis.

### Investigation of the biosynthetic pathway for viomellein by heterologous expression

#### Production of nor-toralactone and semivioxanthin

**Fig 4:**
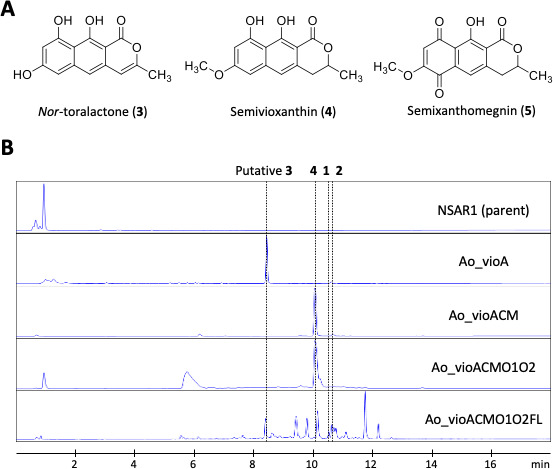
Heterologous expression of the putative viomellein biosynthetic genes in *Aspergillus oryzae*. (**A**) The structures of nor-toralactone (compound **3**), semivioxanthin (compound **4**), and semixanthomegnin (compound **5**). (**B**) HPLC chromatogram of the *A. oryzae* strains expressing the putative viomellein biosynthetic genes. The strains were cultured in minimal medium with appropriate supplements for 5 days. The metabolites were extracted from the cultured mycelia and were then analyzed by HPLC: putative nor-toralactone (compound **3**), semivioxanthin (compound **4**), and viomellein (compounds **1 and 2**).

To confirm the involvement of the *vio* gene cluster in viomellein production and investigate the biosynthetic pathway, we serially reconstructed the *vio* cluster genes (*vioA*, *vioO1*, *vioC*, *vioF*, *vioL*, *vioM*, and *vioO2*) in *Aspergillus oryzae*, a model host for heterologous expression of natural fungal products. First, the strain expressing the PKS *vioA* (Ao_vioA) was constructed. This strain was shown to produce compound **3**, which gave a protonated molecule peak at *m/z* 259.0604 in positive ion mode HRESIMS analysis (Fig. 4; Fig. S5). The molecular formula of compound **3** was predicted to be C_14_H_10_O_5_. We failed to determine the structure of compound **3** by NMR because only broad signals were observed in its NMR spectra. Next, we constructed the strain expressing the putative reductase gene *vioC* and the putative methyltransferase gene *vioM* in addition to *vioA* (Ao_vioACM). This strain was shown to produce semivioxanthin (C_15_H_14_O_5_, compound **4**), which was verified by comparative analysis with a commercial chemical standard (Fig. 4B; Fig. S6). Taking into consideration the putative function of VioC/M and the structure of compound **4**, the product from Ao_vioA was predicted to be *nor*-toralactone (compound **3**), which is known as the precursor of fungal perylenequinone cercosporin (25).

#### Production of vioxanthin

To further estimate the biosynthetic pathway, we introduced the putative oxidase genes *vioO1* and *vioO2* into strain Ao_vioACM by transformation, resulting in the Ao_vioACMO1O2 strain. The main metabolite from this strain was semivioxanthin, which suggested that VioO1 and VioO2 are unable to conduct oxidation of semivioxanthin into semixanthomegnin (compound **5**) or other metabolites (Fig. 4). In addition, an *in vitro* reaction using cell-free homogenates of Ao_vioO1 and Ao_vioO2, expressing *vioO1* and *vioO2*, respectively, further supported that VioO1 and VioO2 are not responsible for oxidation of semivioxanthin leading to semixanthomegnin (Fig. S7).

Another possible reaction is dimerization of semivioxanthin leading to vioxanthin. To verify this reaction, we tried to introduce the putative fasciclin-like protein gene *vioF* and the putative laccase gene *vioL* into strain Ao_vioACM by transformation; however, no transformants were obtained. Alternatively, we tried an *in vitro* reaction system. We constructed a strain (Ao_vioFL) expressing both the *vioF* and *vioL* genes, and semivioxanthin was co-incubated with the cell-free homogenate of Ao_vioFL. Dimerization was confirmed by HRESIMS. After a 4-h reaction, the putative dimerized product vioxanthin, which gave a protonated molecule peak at *m/z* 545.1524 in positive ion mode HRESIMS analysis, was detected in the Ao_vioFL homogenate but not in that of the parental strain (Fig. S8). These results indicated that VioF and VioL are involved in dimerization of semivioxanthin to vioxanthin.

#### Production of viomellein

**Fig 5:**
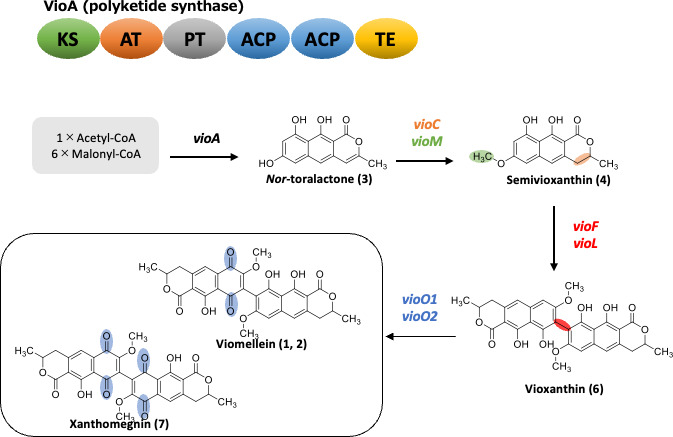
Proposed viomellein biosynthetic pathway.

Finally, we constructed a strain expressing the seven *vio* genes (Ao_vioACMO1O2FL) by introducing the *vioF* and *vioL* genes into strain Ao_vioACMO1O2. LCMS analysis revealed that this strain produced several metabolites including viomellein, xanthomegnin, semivioxanthin, and semixanthomegnin (Fig. 4; Fig. S9). This result showed that these seven *vio* genes are necessary and sufficient to biosynthesize viomellein. Although the final step to produce viomellein remained undetermined, we describe the proposed biosynthetic pathway for viomellein and related compounds in Fig. 5.

### Viomellein production conserved in dermatophytes

Based on sequence similarity search using BLAST, the *vio* cluster is highly conserved among dermatophyte species including *T. mentagrophytes*, *T. tonsurans*, *M. canis*, and *N. gypsea* (Table S2). To assess whether the production of viomellein and related compounds is common among dermatophytes, we examined the metabolite profile for multiple clinical isolates of these dermatophyte species. Most of the isolates produced viomellein (compounds **1** and **2**), vioxanthin (compound **6**), and xanthomegnin (compound **7**) in *T. rubrum*, *T. mentagrophytes*, *T. tonsurans*, and *N. gypsea*, whereas none of the *M. canis* isolates produced viomellein-related compounds (Table 1). Notably, the xanthomegnin production level was higher in *T. rubrum* than in *T. mentagrophytes* and *T. tonsurans*, and these two species produced semivioxanthin (compound **4**). These results indicated that viomellein-related compounds were commonly produced in dermatophytes, except *M. canis*, and the production profile for the compounds appears to be species-dependent.

**TABLE 1:** Viomellein-related compound production capacity of clinical isolates of dermatophytes^*a*^

	Strain	Viomellein	Vioxanthin	Xanthomegnin	Semi-vioxanthin	Semi-xanthomegnin
*T. rubrum*	IFM 62668	+++	++	++	−	−
	IFM 46147	+++	++	+++	−	−
	IFM 64539	+++	+++	+++	−	−
	IFM 61539	+++	++	+++	−	−
	IFM 47628	+++	+++	+++	−	−
	IFM 46262	++	+	++	−	−
	IFM 45896	+++	+++	+++	−	−
	IFM 63292	++	++	++	−	−
	IFM 46035	++	++	++	−	−
	IFM 54833	+++	++	+++	−	−
*T. mentagrophytes*	IFM 47181	++	++	+	+	−
	IFM 46173	++	++	+	+	−
	IFM 48809	+	++	+	+	−
	IFM 55365	++	++	+	+	−
	IFM 47684	++	++	+	+	−
*T. tonsurans*	IFM 62867	+	+	+	−	−
	IFM 61321	++	++	+	+	−
	IFM 62735	++	++	+	+	−
	IFM 54728	++	++	+	+	−
	IFM 55592	−	+	+	−	−
*M. canis*	IFM 61533	−	−	−	−	−
	IFM 53932	−	−	−	−	−
	IFM 63748	−	−	−	−	−
	IFM 54199	−	−	−	−	−
	IFM 55364	−	−	−	−	−
*M. gypseum*	IFM 62342	+	+	−	−	−
	IFM 62747	+	+	−	−	−
	IFM 62744	++	++	+	−	−
	IFM 56886	++	++	++	−	−
	IFM 48629	+	+	−	−	−

^*a*^ Production level for the conpounds in each strain was measured by peak area in HPLC analysis (λ=254 nm). +++ : >1,000; ++ : >100; + : >10; − : <10.

The minimum inhibitory concentrations (MICs) of viomellein (compounds **1** and **2**) and related compounds were determined against *S. aureus* and the pathogenic fungus *C. albicans*. Viomellein showed the highest activity against *S. aureus* and *C. albicans* (MICs: 0.78 and 6.25 µg/mL, respectively), while the activity of semivioxanthin (compound **4**) and semixanthomegnin (compound **5**) against *S. aureus* was also potent (Table 2).

**TABLE 2:** MICs for *S. aureus* and *C. albicans^a^*

Compound	MIC [µg/mL]	
	*S. aureus*	*C. albicans*
Viomellein	**0.78**	6.25
Xanthomegnin	25	12.5
Semi-vioxanthin	1.56	>100
Semi-xanthomegnin	1.56	50
Chloramphenicol	0.39	NA
Miconazole	NA	<0.12

^*a*^ NA, not assayed.

## DISCUSSION

Fungi have the potential to produce many secondary metabolites in their natural habitats, such as soil, aquatic environments, and within host organisms, including plants, insects, and animals. Several compounds with antibacterial activity have been discovered, with penicillin being the most famous fungal metabolite. However, it remains to be understood practically why fungi produce antibacterial compounds. Such antibiotic metabolites may protect the producer fungi from other microorganisms in their surroundings and, if so, these compounds may be vital for the establishment of infection. Based on this hypothesis, researchers have long sought the antibacterial compounds produced by pathogenic dermatophytes, the causative agent of skin infections. In the present study, we revisited this area of research and rediscovered the pigment viomellein as an antibacterial compound. We also identified the biosynthetic genes that had previously remained uncharacterized. Our insight into the chemical and genetic basis of this antibacterial compound may help elucidate the interactions between dermatophytes and microbial communities on the skin surface and will contribute to further understanding of the infection process of dermatophytes.

Antibacterial naphthopyranone dimer compounds, such as viomellein, xanthomegnin, and vioxanthin, have been detected in cereals and animal feed (26–28), as well as in epidermal clinical materials (22). Xanthomegnin was originally found to be produced by *T. megnini* (16), and other dermatophytes, such as *T. rubrum*, *T. violaceum*, and *M. cookei* were also reported to produce this compound (17, 18, 29). Apart from dermatophytes, the producer fungi of xanthomegnin and related compounds have been reported and include *Penicillium viridicatum*, *P. freii*, *P. cyclopium*, *A. ochraceus*, *A. elegans*, *A. flavus*, *A. sulphureus*, *A. melleus*, and *Aspergillus* sp. FM242 (23, 30–35). A BLAST search revealed that PKS VioA orthologues (>60% identity) were present in several *Penicillium* and *Aspergillus* species, such as *P. freii*, *P. viridicatum, A. steynii, A. melleus*, and *Aspergillus* sp. FM242, which was consistent with the reported metabolite production by these species. Despite the wide distribution of such antibacterial compounds, genetic characterization of their biosynthetic pathways has been lacking. Therefore, identification of the *vio* cluster provides a genetic explanation for the diversity of these structurally complex and activity-rich naphthopyranone compounds in fungi. In addition, this genetic insight paves the way to assessing the ecological and pathological functions of these compounds.

The gene cluster involved in the biosynthesis of viriditoxin, a dimeric naphthopyranone related to vioxanthin, was previously predicted by comparing the genome sequences of the producer fungi (24, 36). The laccase gene within the gene cluster in *A. viridinutans* was proven to encode a coupling enzyme (Av-VilL), which catalyzed the dimerization of semiviriditoxin resulting in viriditoxin production, as well as the conversion of semivioxanthin to vioxanthin. Accordingly, we demonstrated that *T. rubrum* VioL, the orthologue of Av-VilL, catalyzed the dimerization of semivioxanthin into vioxanthin in heterologously expressed *A. oryzae*. Interestingly, the production of the monomeric substrates for viomellein, such as semivioxanthin and semixanthomegnin, was undetectable in the *T. rubrum* isolates (Table 1). This suggested that the dimerization step proceeds rapidly. Oxidation reactions by VioO1 and VioO2 generate different products depending on their degree of oxidation, creating chemical diversity, which may serve as a survival strategy in interactions with other organisms.

An antimicrobial activity test revealed that viomellein had the lowest MIC (0.78 µg/mL) against *S. aureus* among the related compounds, which is as effective as chloramphenicol. In several previous studies, the bioactivities of the viomellein-related compounds have been evaluated. Among viomellein, xanthomegnin, and vioxanthin, viomellein and vioxanthin showed low IC_50_ values (1.6–3.2 µM) against *S. aureus* and MRSA, whereas the IC_50_ values against A549, human lung carcinoma cells, ranged from 42.0 to 77.1 µM (37). Kumla et al*.* reported that vioxanthin had the most potent activity against *Enterococcus faecalis* and *S. aureus* (MICs: 1–2 and 2 µg/mL, respectively), whereas the MICs of xanthomegnin were 32 and 16–32 µg/mL, and those of viomellein were 8 and 4–8 µg/mL, respectively (33). Viomellein also exhibited potent anti-dormant mycobacterial activity (MIC against *Mycobacterium bovis* BCG: 1.56 µg/mL), whereas xanthomegnin showed weak activity against *M. bovis* BCG with an MIC of 50 µg/mL (38). In summary, viomellein exerts high antibacterial activity, and xanthomegnin exerts relatively low antibacterial activity.

Viomellein was also reported to show cytotoxicity against the L5178Y mouse lymphoma cell line (IC_50_: 5.0 µM) and against the A2780 human ovarian carcinoma cell line (IC_50_: 5.3 µM), but was inactive against the human Jurkat T and Ramos B cell lines (39). Semivioxanthin showed no antiproliferative activity (>40 µM) against four different types of cancer cell lines, HT1080, PC3, Jurkat, and A2780 (35). Xanthomegnin has been reported to be a potent inhibitor of inducible nitric oxide synthase with an IC_50_ value of 5.5 µM (21). These assays revealed that viomellein is highly bioactive against host cells. Aside from the effects on the skin microbiota, these properties may aid the progression of pathogenesis. The potential involvement of these metabolites in the pathogenesis of dermatophytes is an important topic for future research.

## MATERIALS AND METHODS

### Strains

The dermatophyte strains used in this study were provided by the National Bio-Resource project (https://nbrp.jp/) (Table S3). Bacterial and fungal strains used for bioassays, including *E. coli* ATCC 25922, *B. subtilis* PC1219, *S. aureus* 209P, *Micrococcus luteus* IFM 2066, *Candida albicans* IFM 62681, and *A. niger* IFM 62678, were obtained from Chiba University Medical Mycology Research Center through the National Bio-Resource Project, Japan. *S. epidermidis* NBRC12933 was obtained from the NITE Biological Resource Center.

The *A. oryzae* NSAR1 strain (40) was used for heterologous expression experiments. The *A. oryzae* NSAR1 strain is an auxotrophic quadruple mutant (*ΔargB*, *sC*˗, *adeA*˗, and *niaD*˗), which together with a pyrithiamine resistance marker (*ptrA*) allows for five different selections. *E. coli* DH5α (Takara Bio, Shiga, Japan) was used for plasmid construction.

### Media

SDA (BD Bioscience) and PDA (BD Bioscience) were used for fungal culture. For liquid culture, Sabouraud dextrose broth (SDB) (BD Bioscience) was used. The temperature for *Trichophyton* culture was set at 30°C, unless otherwise specified. Spore formation was promoted at 28°C using 1/10 SDA supplemented with 500 µg/mL cycloheximide and 50 µg/mL chloramphenicol (Fujifilm Wako Pure Chemical Corporation).

### Chemicals

The standard compounds, viomellein, xanthomegnin, semivioxanthin, and semixanthomegnin were commercially obtained (Santa Cruz Biotechnology, Dallas, TX, USA).

### Bioassay

For the diffusion assays using agar plugs, *T. rubrum* and *T. mentagrophytes* strains were grown on SDA and PDA at 30°C for 7 days. Agar plugs (diameter: 5 mm) were cut from the plate media and placed onto the LB agar containing precultured bacteria. The plates were incubated at 30°C for 1 or 2 days. Penicillinase solution (Tokyo Chemical Industry Co., Ltd, Tokyo, Japan) was used at a dilution of 1/1,000 in the media.

For the paper disc assays, fungal strains were grown on SDA at 30°C for 3 weeks. The cultured plates were then lyophilized, and 20 mL of methanol was added and shaken vigorously. The supernatant was then collected and concentrated 10-fold to make an extract. Next, 10 µL of the extract was added to a 6 mm diameter paper disc, and the disc was air-dried for 5 min. The sample-soaked paper discs were placed onto the test medium and adhered tightly, and the plates were incubated for 1–2 days before checking for halo formation.

The test media were prepared as follows. The bacteria (*E. coli*, *S. aureus*, *B. subtilis*, and *M. luteus*) and fungi (*C. albicans*) were precultured in LB and SDB, respectively, overnight. Then, a 1/200 to 1/5,000 vol of the preculture was added to the agar medium before solidification. For *A. niger*, the conidia were added to LB agar in the same manner.

### Purification of antibacterial compounds

*T. rubrum* was cultured in SDA for 3 weeks at 30°C. For purification of the compounds with bioactivity, 100 plates were used to collect the crude extract. The culture plates were lyophilized and extracted with 5 L of methanol and then 5 L of ethyl acetate. The extracts were combined, evaporated, and then separated into ethyl acetate and water. The ethyl acetate (upper) phase was collected, concentrated *in vacuo*, and fractionated by octadecylsilyl silica gel (ODS) flash chromatography, which yielded 20%, 40%, 60%, 80%, and 100% methanol fractions, and a CMW (chloroform:methanol:water = 7:3:0.5) fraction. The 80% methanol fraction was further purified using a 1260 Infinity LC system (Agilent Technologies, Inc., CA, USA) with a COSMOSIL Packed Column 5C_18_-AR-II column (ϕ10 mm × 250 mm; Nacalai Tesque, Kyoto, Japan). The high-performance liquid chromatography (HPLC) analytical conditions were an isocratic elution of 48% acetonitrile containing 0.5% acetic acid for 40 min at a flow rate of 4 mL/min. After each step of fractionation, bioactivity was examined by a bioassay against *B. subtilis*. HRESIMS analysis was performed using UPLC-SYNAPT G2 HDMS (Waters, Milford, MA, USA).

### RNA-sequencing analysis

The conidia of *T. rubrum* IFM 64539 were inoculated onto the surface of an SDA plate and then incubated at 30°C for 9 days. From the colonies, 10 agar plugs were collected and flash frozen. The frozen mycelia were ground to a powder with a mortar and pestle. The mycelial powder was then added to TRIzol solution (Invitrogen). The Purelink RNA Mini Kit (Thermo Fisher Scientific) and Zymoclean Gel RNA Recovery Kit (ZYMO Research, CA, USA) were used to purify the total RNA. In the case of liquid culture, the conidia were inoculated into SDB at a final concentration of 10^5^ conidia/mL and rotate-cultured at 150 rpm at 30°C for 12 days. RNA extraction was performed as for plate culture.

Library preparation and paired-end sequencing were performed by Novogene (Beijing, China). The read data were trimmed and mapped to the reference genome of *T. rubrum* CBS 118892, and then TPM were calculated using CLC genomics workbench (QIAGEN, Hilden, Germany).

### Constructing plasmids for heterologous expression in *A. oryzae*

#### Vector preparation

For expression plasmid construction, pUARA2 (41), pUSAN (42), pAdeAN (42), and pPTR I (Takara) were used. pUARA2 contains the *argB* gene as a selective marker and two amylase promoter-terminator cassettes (P*amyB*–T*amyB* and P*amyB*–T*amyA*). pUARA2 has a multicloning site between P*amyB* and T*amyB* and between P*amyB* and T*amyA*, allowing gene insertion. pUSAN has a methionine-requiring complementary gene (*sC*) and two amylase promoter-terminator cassettes (P*amyB*–T*amyB* and P*amyB*–T*amyA*) derived from pUARA2. pAdeAN has an adenine-requiring complementary gene (*adeA*) and one P*amyB*–T*amyA* cassette derived from pUARA2. pPTRI has a pyrithiamine resistance gene (*ptrA*). The P*amyB*–T*amyB*–P*amyB*–T*amyA* region of pUARA2 was inserted into pPTRI as follows. pPTRI was treated with *Pst*I and *Kpn*I, and the PCR-amplified P*amyB*–T*amyB*–P*amyB*–T*amyA* cassette was inserted into pPTRI using the In-Fusion HD Cloning Kit (Takara) to obtain pPTRIN.

#### Gene fragment preparation

DNA fragments for each gene were generated by reverse transcription-PCR using the PrimeScript II 1st strand cDNA Synthesis Kit (Takara). The total RNA of *T. rubrum* strain IFM 64539 was used as the template for RNA sequencing.

#### Plasmid construction

pUARA2, pUSAN, pAdeAN, and pPTRIN were treated with appropriate restriction enzymes, and each gene fragment was inserted into the restriction site(s) of the vector using the In-Fusion HD Cloning Kit. The *vioA* fragment was inserted into the *Kpn*I and *Cla*I sites of pUARA2 to obtain *vioA*/pUARA2. Two fragments *vioC* and *vioM* were inserted into the *Kpn*I and *Cla*I sites of pUSAN, respectively, to obtain *vioC+vioM*/pUSAN. Two fragments *vioL* and *vioF* were inserted into the *Kpn*I and *Nhe*I sites of pPTRIN, respectively, to obtain *vioF+vioL*/pPTRIN. *vioO1* and *vioO2* fragments were inserted into the *Nhe*I site of pAdeAN to obtain *vioO1*/pAdeAN and *vioO2*/pAdeAN, respectively. The P*amyB-vioO1*-T*amyB* cassette was amplified by PCR using *vioO1*/pAdeAN as template and was inserted into the *Xba*I site of *vioO2*/pAdeAN to obtain *vioO1+vioO2*/pAdeAN. In addition to colony PCR after *E. coli* transformation, DNA sequencing of each purified plasmid confirmed the absence of mutations in the gene region, and the plasmids were used as expression plasmids for heterologous expression. Finally, six expression plasmids, *vioA*/pUARA2, *vioC+vioM*/pUSAN, *vioF+vioL*/pPTRIN, *vioO1+vioO2*/pAdeAN, *vioO1*/pAdeAN, and *vioO2*/pAdeAN, were constructed (Fig. S10). The primers used are shown in Table S4.

### Transformation of *A. oryzae*

*A. oryzae* NSAR1 or the related strains were cultured in 60 mL of sugar soy sauce medium (50 g/L white sugar and 50 g/L soy sauce) at 30°C, 120 rpm, for 3 days (43). The harvested mycelia were washed with distilled water and treated with lysing enzyme (5 mg/mL) and yatalase (5 mg/mL) in sodium phosphate buffer (pH 6.0) with 0.8 M NaCl at 30°C for 1–3 h to generate protoplast cells. The mycelia were removed by passing through a 40 µm pore cell strainer. The protoplasts were collected by centrifugation (3,000 rpm, 3 min), and the supernatant was discarded. The protoplasts were washed with 0.8 N NaCl three times, and then suspended in Solution I (0.8 N NaCl, 10 mM CaCl_2_, and 10 mM Tris-HCl [pH 8.0]). Transformation was performed by the protoplast-polyethylene glycol (PEG) method, as described previously (44).

### Metabolite analysis

Unless otherwise specified, the above-mentioned 1260 Infinity LC system with a Poroshell 120 EC-C18 column (ϕ3.0 mm × 100 mm, particle size 2.7 µm; Agilent) was used for metabolite analysis. The HPLC analytical condition was gradient elution in 5–100% acetonitrile in water containing 0.5% acetic acid over 18 min.

### *In vitro* reaction

Each *A. oryzae* strain expressing genes of interest was cultured in the same manner as for metabolite analysis. The mycelia were collected and bead-disrupted in 50 mM sodium phosphate buffer. After centrifugation, the supernatant was transferred to a new tube and used as the enzyme extract. Then, 3 µL of semivioxanthin or semixanthomegnin prepared in 1 mg/mL DMSO was added to 300 µL of enzyme extract and incubated at 30°C for a specified time. The reaction solution was passed through an ODS column to remove impurities and analyzed by HPLC or LCMS.

### Construction of the *vioA* deletion mutant

#### Preparing a gene deletion fragment

Genomic DNA of *T. rubrum* IFM 46035 was extracted from the mycelia, according to a method by Girardin and Latge (45), with minor modifications. Fragments of the flanking regions of the *vioA* (TERG_02850) open reading frame were amplified by PCR with primer pairs P1–P4 and P5–P6 using the genomic DNA as a template (Table S4). The fragment of the 5ʹ-untranslated region (UTR) (2.38 kb) was digested with *Spe*I/*Apa*I and cloned into pAg1-TinCYP51B/T (46). The 3ʹ-UTR (2.54 kb) was then digested with *Bam*HI/*Kpn*I and cloned into the same plasmid, resulting in pAg1-vioA/R containing an *nptII* cassette. To inactivate the *Apa*I sites within the fragment, overlap extension PCR was performed using PrimeSTAR HS or PrimeSTAR GXL DNA polymerases (Takara Bio) with corresponding primers (Table S4). The 5ʹ-UTR + nptII + 3ʹ-UTR fragment for gene replacement was amplified by PCR using primers P7–P8 (Table S4).

#### Ribonucleoprotein (RNP) complex preparation for CRISPR

The 23-nucleotide target-specific nucleotide sequences around the translation initiation and termination codons of the *vioA* gene (5′-GACCTCGTGGCCTTGTACCAAGG-3′ [− strand]; 5′-TGCATACGGGGAAGATAACTGGG-3′ [+ strand]) were manually chosen to synthesize Alt-R crRNA (Integrated DNA Technologies). Two guide RNAs (gRNA1 and gRNA2) were prepared by mixing equal amounts of Alt-R crRNA and Alt-R tracrRNA (Integrated DNA Technologies) in IDT Duplex Buffer (30 mM HEPES, pH 7.5, 100 mM potassium acetate; Integrated DNA Technologies), heating to 95°C, and slowly cooling to room temperature. An equal amount of CRISPR-Cas nuclease (HiFi Cas9 Nuclease V3, Integrated DNA Technologies) was added to the gRNA and incubated for 20 min at room temperature, resulting in RNP complexes, Cas9/gRNA1 RNP and Cas9/gRNA2 RNP.

#### Transformation of *T. rubrum*

Protoplast formation of *T. rubrum* IFM 46035 was performed according to the method of Yamada et . (47), with minor modifications. Briefly, the fragment for gene replacement (2.5–5.0 µg) and two RNP complexes (55.0–57.0 nM) were introduced into the protoplasts by the PEG-mediated method. Then, the protoplasts were gently spread onto SDA supplemented with 1.2 M D-sorbitol and 1% yeast extract containing 250 µg/mL G418 (Fujifilm Wako Pure Chemical Corporation). After incubation, a large number of putative G418-resistant transformants appeared on the selective medium, and 25 were chosen at random and analyzed using molecular biological methods. Through direct colony PCR with primer pairs P12–P13 and P9–P10 (Table S4), candidate strains were selected. The strains were further verified through PCR against extracted genomic DNA and Southern hybridization analysis. For Southern hybridization, approximately 5 µg of the total DNA was digested with *Eco*RI, separated by electrophoresis on 0.8% (wt/vol) agarose gels, and transferred onto Hybond-N+ membranes (Cytiva). The probe was generated by PCR using primers P4 and P9 (Table S4). Southern hybridization was performed with an ECL Direct Nucleic Acid Labeling and Detection System (Cytiva), according to the manufacturer’s instructions.

## Data Availability

The data sets presented in this study are available in NCBI/ENA/DDBJ under BioProject no. PRJDB20304.

## SUPPLEMENTAL MATERIAL

Supplemental figures aem.02431-24-s0001.pdf Figures S1 to S10.

Supplemental tables aem.02431-24-s0002.xlsx Tables S1 to S4.

ASM does not own the copyrights to Supplemental Material that may be linked to, or accessed through, an article. The authors have granted ASM a non-exclusive, world-wide license to publish the Supplemental Material files. Please contact the corresponding author directly for reuse.
