# Supplementary material for: Rediscovery of viomellein as an antibacterial compound and identification of its biosynthetic gene cluster in dermatophytes
Source: Appl Environ Microbiol. 2025 Apr 8;91(5):e02431-24. doi: 10.1128/aem.02431-24 (PMC12093964; doi:10.1128/aem.02431-24)
Supplement: Supplemental figures — Figures S1 to S10. [file aem.02431-24-s0001.pdf]

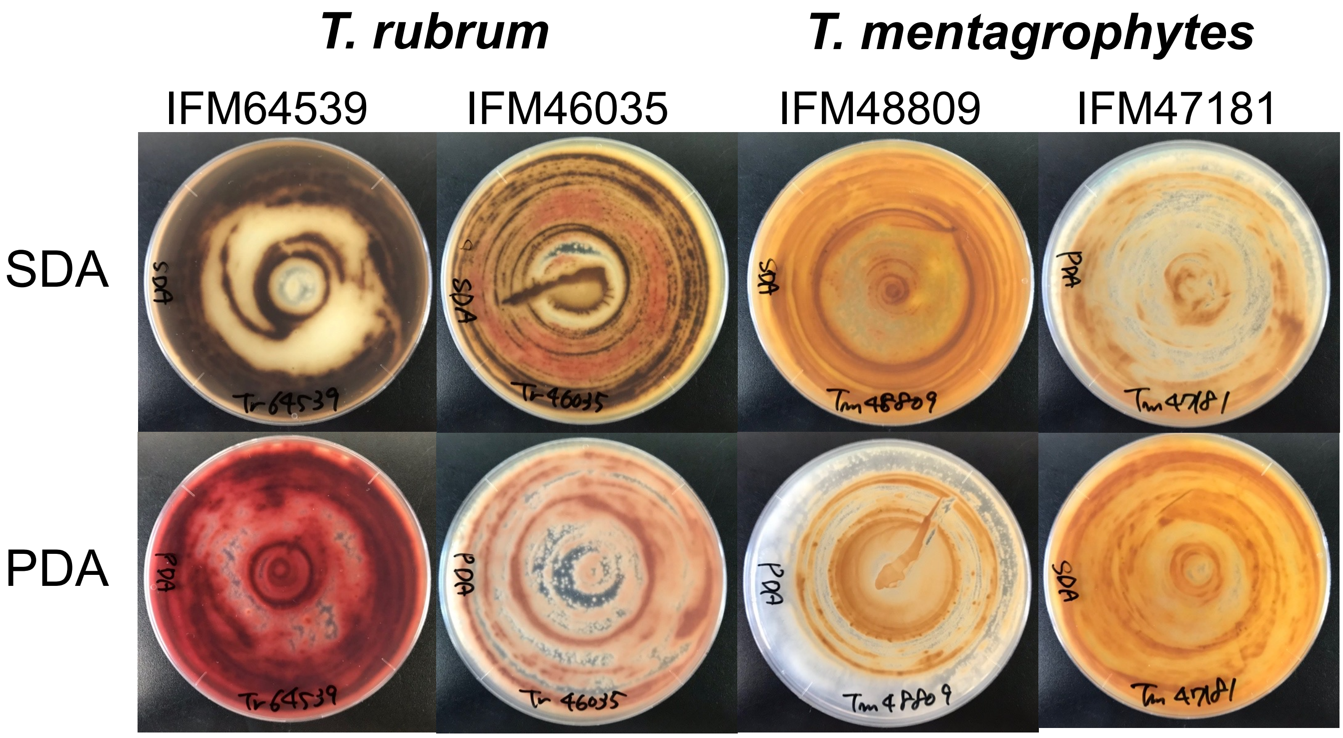

Fig. S1 Colony growth of *T. rubrum* and *T. mentagrophytes* on plate media

The conidia of *T. rubrum* and *T. mentagrophytes* were inoculated onto SDA and PDA, and spread over the entire surface of the plate. The plates were incubated at 30° C for 3 weeks.

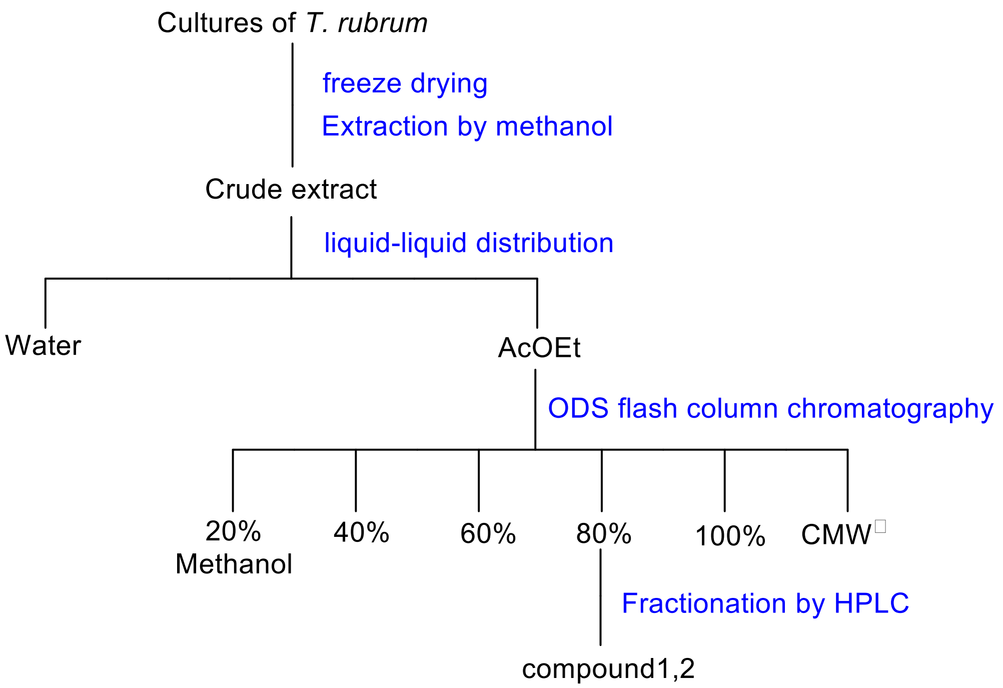

□CMW chroloform:methanol:water = 7:3:0.5

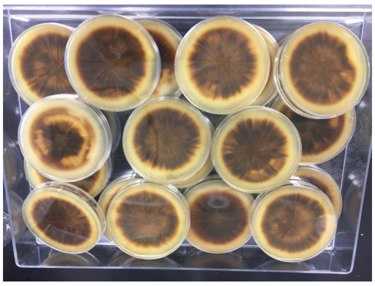

Cultures of T. rubrum

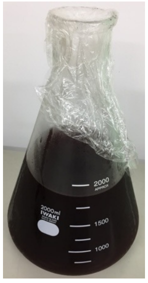

Crude extract

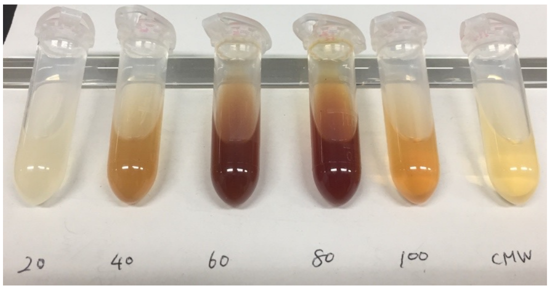

Fractions of column chromatography

Fig. S2 Scheme of activity-guided fractionation

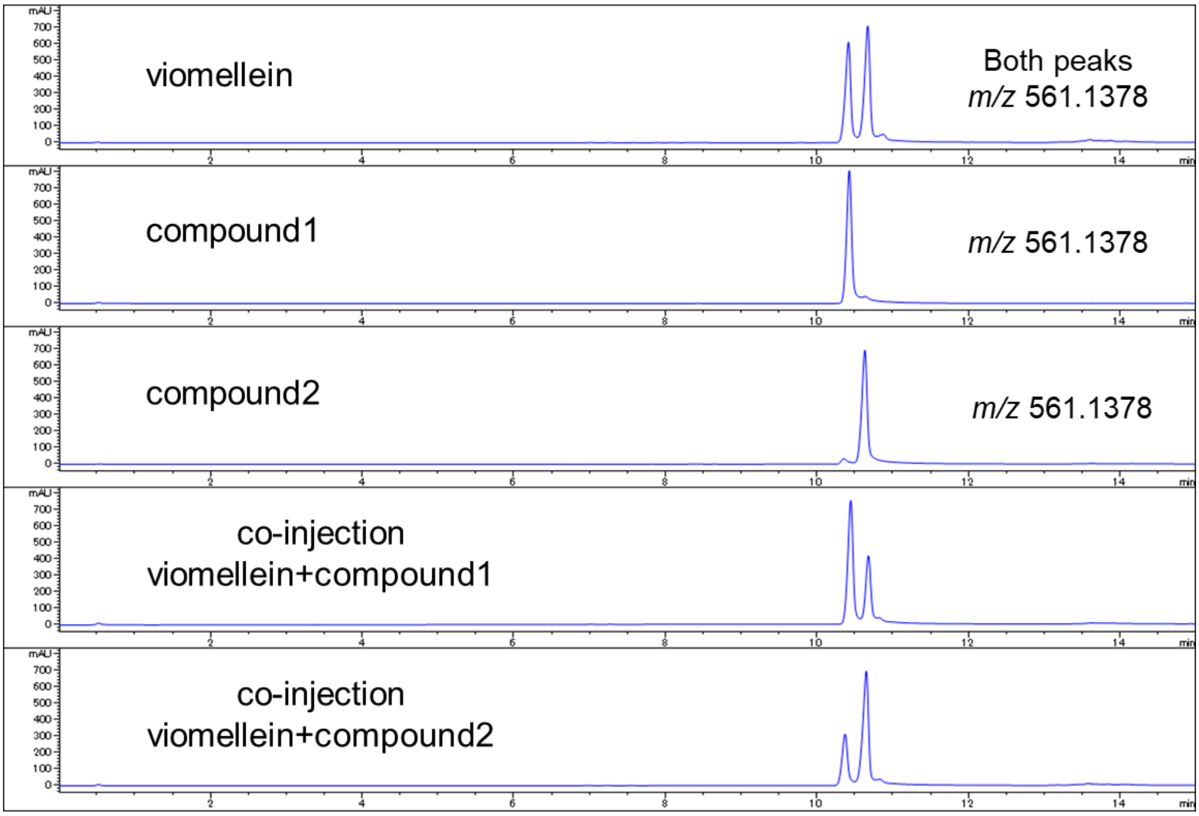

Fig. S3 Chromatogram of standard viomellein and purified viomellein derived from HPLC analysis

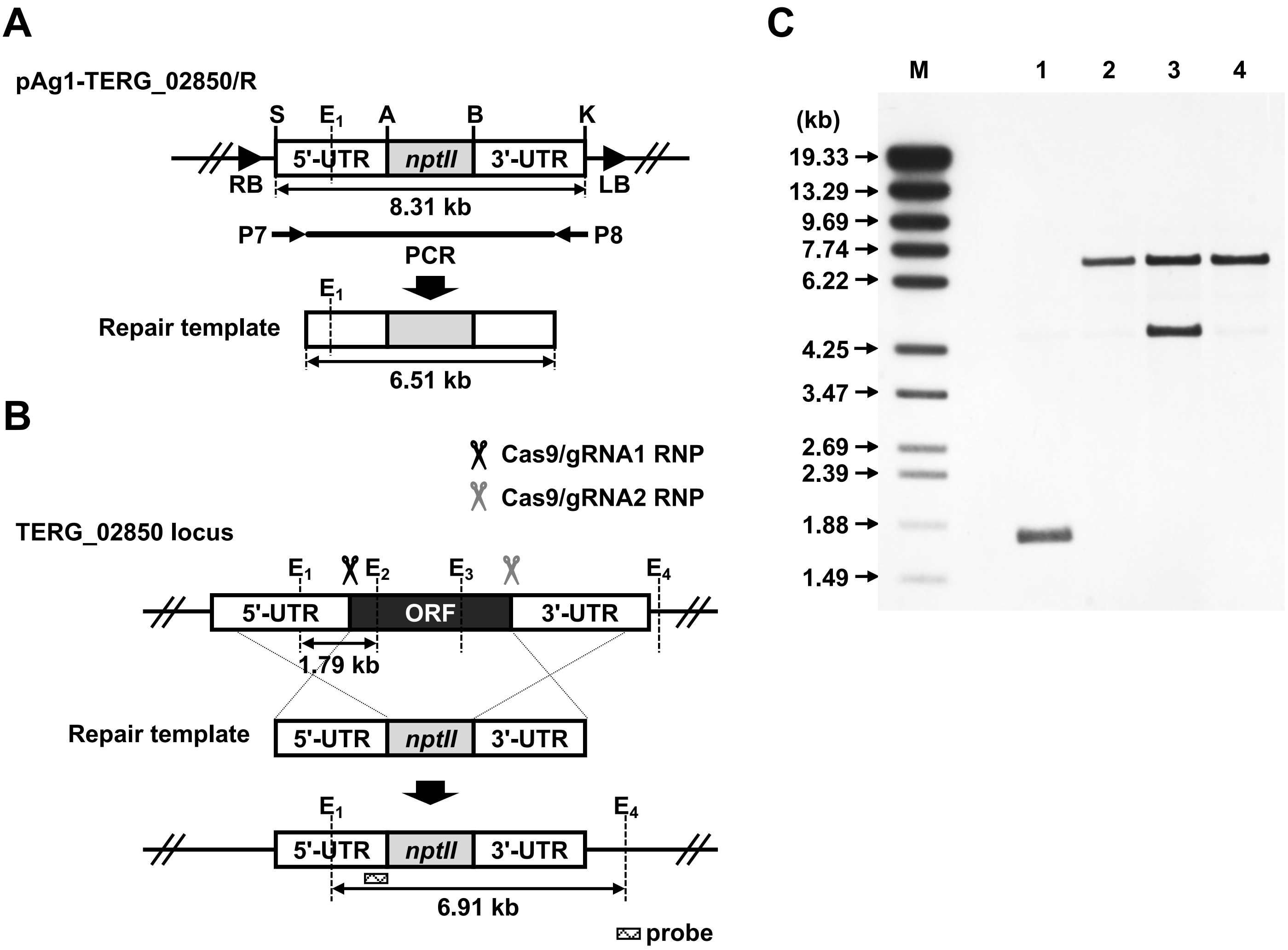

Fig. S4 Construction of the *vioA* gene deletion mutant

A. Schematic representation of the repair template. The binary vector pAg1-TERG_20850/R contained a *nptII* cassette and the 5'- and 3'-flanking regions of the *vioA* gene. pAg1-TERG_02850/R was used to PCR amplify the repair template fragment using the P7-P8 primers. S, A, B, and K indicate the restriction enzyme cleavage sites for *Spe*I, *Apa*I, *Bam*HI, and *Kpn*I, respectively. **B**. Schematic representation of the deletion of the *vioA* gene. The TERG_02850 (*vioA*) locus was replaced by the repair template by homologous recombination as shown. The cleavage sites for CRISPR/Cas9 are shown. E_1–E_4 indicate the *Eco*RI cleavage sites. **C**. Southern blotting analysis. Genomic DNA from the parental strain and each transformant was digested with *Eco*RI and separated by electrophoresis on 0.8% (w/v) agarose gels. Lane 1: parental strain; lanes 2 to 4: transformant-1, -3, and -5; M: DNA standard fragments (λ-*Eco*T14I/*Bgl*II digest). DNA standard fragment sizes are shown on the left. The fragment of 520 bp was amplified by the P9-P4 primers and used as a hybridization probe.

A

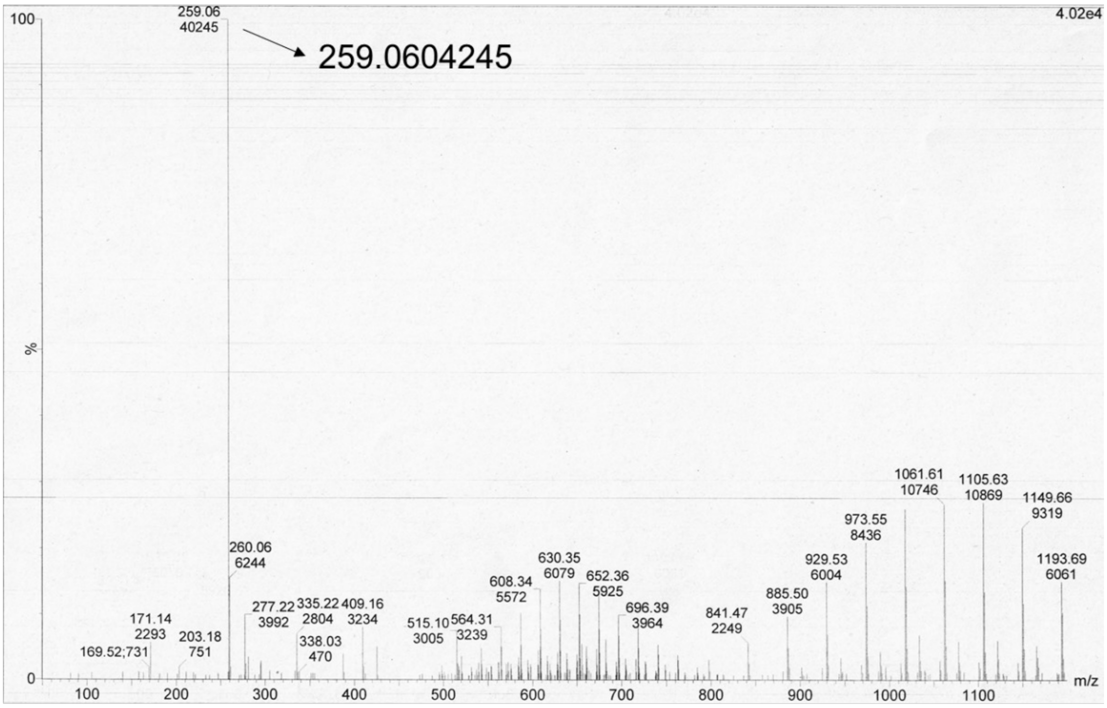

B

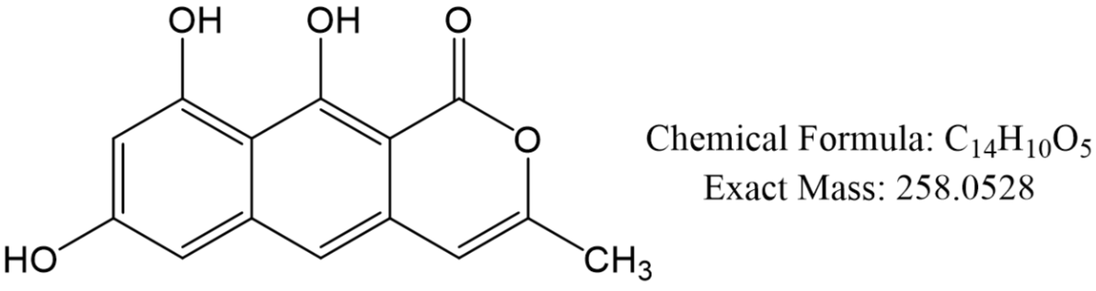

nor-toralactone

Fig. S5 Exact molecular mass of the metabolite from strain Ao_vioA

A. Mass spectrum of the Ao_vioA metabolite. **B**. Nor-toralactone (**3**).

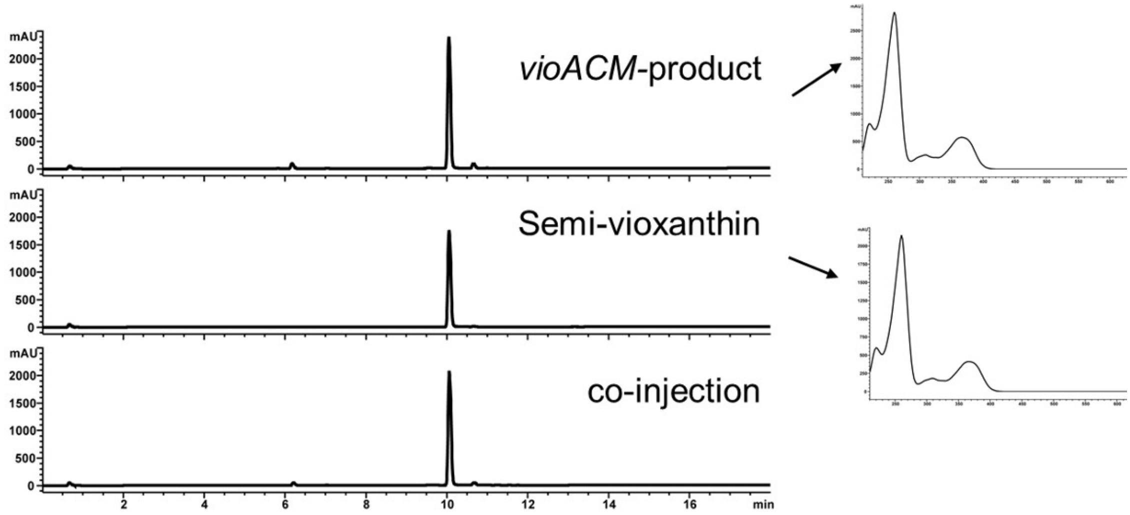

Fig. S6 Confirmation of semivioxanthin production in strain Ao_vioACM

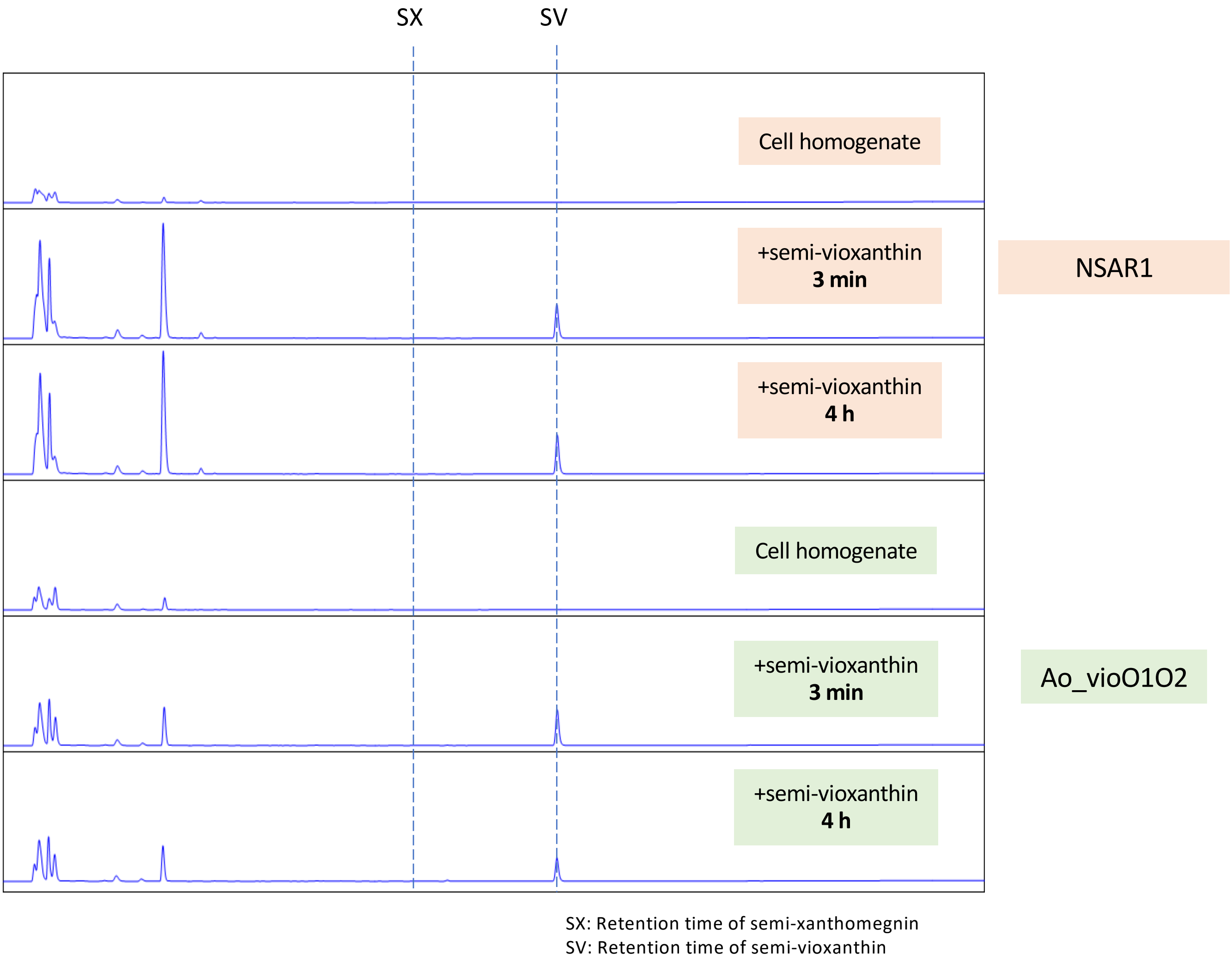

Fig. S7 *In vitro* reaction of Ao_vioO1O2 with semivioxanthin

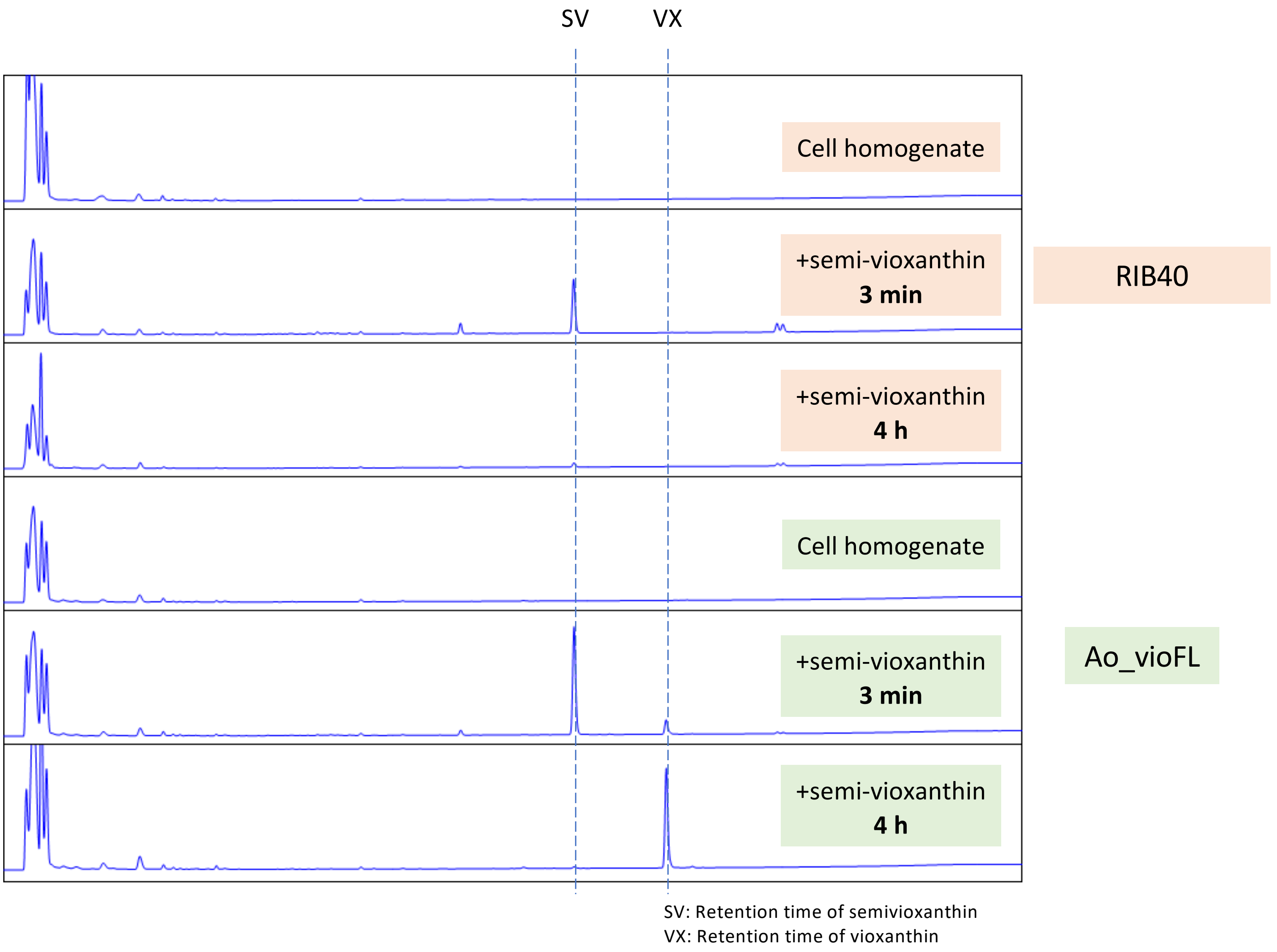

Fig. S8 *In vitro* reaction of Ao_vioFL with semivioxanthin

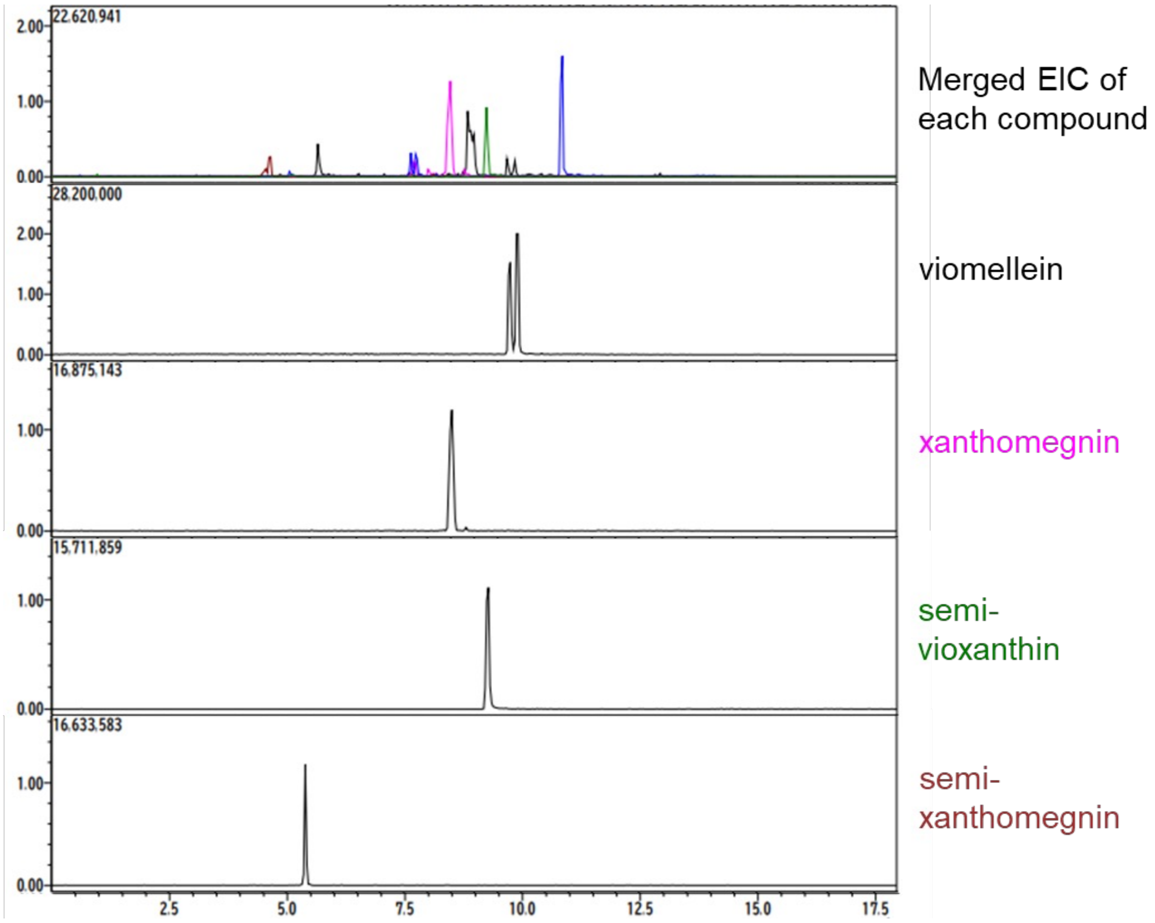

Fig. S9 Mass chromatograms of Ao_vioACMO1O2FL.
The extract of Ao_vioACMO1O2FL was analyzed by negative ion mode LCMS analysis. The second, third, fourth, and fifth chromatogram shows an extracted ion chromatogram at *m/z* 559.13, 573.11, 273.08, and 287.06, respectively. The first chromatogram shows merged extracted ion chromatogram of each compound.

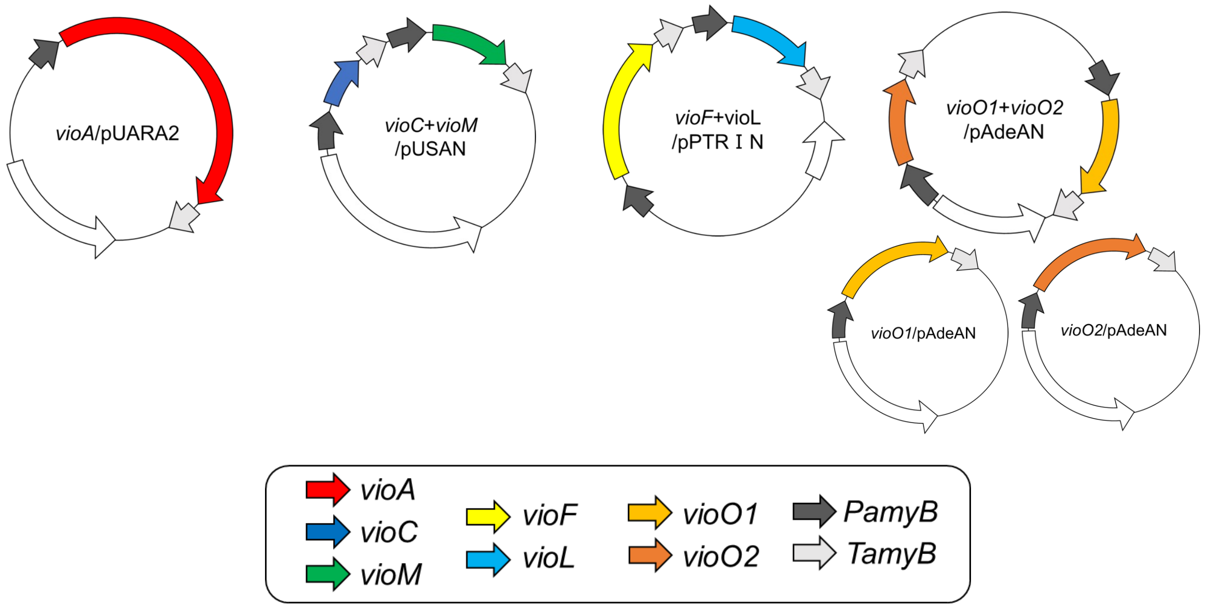

Fig. S10 The plasmids used for heterologous expression experiments in this study
